# Exploring resilience in rural GP registrars – implications for training

**DOI:** 10.1186/s12909-015-0399-x

**Published:** 2015-07-02

**Authors:** Lucie Walters, Caroline O. Laurence, Joanne Dollard, Taryn Elliott, Diann S. Eley

**Affiliations:** 1 Flinders University Rural Clinical School, PO Box 3570, Mount Gambier, SA Australia; 2 Discipline of General Practice, The University of Adelaide, Adelaide, SA 5005 Australia; 3 Adelaide to Outback GP Training Program, 183 Melbourne Street, North Adelaide, SA 5006 Australia; 4 School of Medicine, The University of Queensland, 288 Herston Road, Herston, QLD 4006 Australia

**Keywords:** Resilience, Rural, GP Registrars, Medical education, Vocational training, Rural generalism

## Abstract

**Background:**

Resilience can be defined as the ability to rebound from adversity and overcome difficult circumstances. General Practice (GP) registrars face many challenges in transitioning into general practice, and additional stressors and pressures apply for those choosing a career in rural practice. At this time of international rural generalist medical workforce shortages, it is important to focus on the needs of rural GP registrars and how to support them to become resilient health care providers. This study sought to explore GP registrars’ perceptions of their resilience and strategies they used to maintain resilience in rural general practice.

**Methods:**

In this qualitative interpretive research, semi-structured interviews were recorded, transcribed and analysed using an inductive approach. Initial coding resulted in a coding framework which was refined using constant comparison and negative case analysis. Authors developed consensus around the final conceptual model. Eighteen GP registrars from: Australian College of Rural and Remote Medicine Independent Pathway, and three GP regional training programs with rural training posts.

**Results:**

Six main themes emerged from the data. Firstly, rural GP registrars described four dichotomous tensions they faced: clinical caution versus clinical courage; flexibility versus persistence; reflective practice versus task-focused practice; and personal connections versus professional commitment. Further themes included: personal skills for balance which facilitated resilience including optimistic attitude, self-reflection and metacognition; and finally GP registrars recognised the role of their supervisors in supporting and stretching them to enhance their clinical resilience.

**Conclusion:**

Resilience is maintained as on a wobble board by balancing professional tensions within acceptable limits. These limits are unique to each individual, and may be expanded through personal growth and professional development as part of rural general practice training.

## Background

Resilience can be defined as the ability to rebound from adversity and overcome difficult circumstances [1]. General Practice (GP) registrars have a challenging role as they transition from the tertiary hospital environments of their early postgraduate employment to the community contexts of primary and more independent secondary care. This transition involves learning to manage clinical uncertainty, patient expectations, and junior doctors becoming more independently accountable for their medical decisions. A lack of tolerance for uncertainty has been linked with burnout, anxiety and poor job satisfaction in doctors [2, 3]. For those choosing a career in rural practice, additional stressors and pressures apply including the potential for professional and social isolation.

Australia is a highly urbanised country with more than 89 % of people living within 50 km of the coast, and 70 % of people living in cities greater than 1 million people. Due to rural medical workforce shortages, the Australian Commonwealth Government has funded 50 % of GP training posts in rural and remote areas. It is Australia’s unique geography and sparsely populated interior which make addressing the rural workforce problematic. Distances are great between the small towns and rural centres with low population density limiting capacity for civic amenities and sustainable secondary and tertiary health services.

It is important to study the resilience of rural GP registrars as rural training pathways are being developed and expanded in many countries to address rural medical workforce shortages [4–7]. We have a social responsibility to GP registrars to ensure these pathways do not impact negatively on their wellbeing. At a practical level it is likely that GP registrars who are better able to manage the challenges of rural generalism will potentially have higher retention rates in rural practice, increasing the success of these training pathways [8].

Two recent studies undertaken in Australia [2, 9] found moderately high resilience in GP registrars, however neither study explored how this cohort achieved or maintained their resilience. Effective resilience promoting strategies in the GP workforce have been demonstrated to include: accepting personal limitations; balancing and prioritising; and having positive personal and professional relationships [10]. Australian doctors working in challenging areas have also been demonstrated to have greater resilience when they: have deep respect for the people they service; engage intellectually with their clinical work; celebrate small gains in individual patient outcomes; and can control their work hours [11]. The aims of this study were to explore GP registrars’ perceptions of their resilience and strategies they used to maintain resilience.

## Methods

As part of a larger study, GP registrars from the Australian College of Rural and Remote Medicine Independent pathway and three regional training providers funded by GPET completed self-report questionnaires on their personality and resilience [9]. Participants were asked if they would consent to a follow-up interview. Ethics approval was obtained from Human Research Ethics Committees at The University of Queensland Medical [#2010001618], University of Adelaide [#H-047-2011] and Flinders University [#5134].

A semi-structured interview guide was developed de novo by the investigators. The interview guide covered several domains: including three questions about resilience - the focus of this publication. These questions sought to explore registrars’ perceptions of the stressors they faced, perceptions of their own resilience and strategies they used to maintain resilience in rural general practice training.

A purposive sample of GP registrars from the original cohort of 479 were invited to participate, representing the diversity of the group in terms of age, gender, country of origin, marital status, location of partner and resilience scores. Interviews were conducted either by telephone or face-to-face where possible. Interviews ranged from 28 to 64 min, were digitally recorded, transcribed verbatim, and entered into NVivo 10 (QSR International Pvt Ltd). Interviews and analysis continued until saturation of themes occurred.

Thematic analysis was conducted using a grounded theory approach where initial codes, categories and finally themes emerged from the data through an inductive approach. Constant comparison was used throughout coding, checking for similarities and differences between codes. Initial coding was performed by JD and TE. The coding framework was reviewed by the wider research team and critical discourse resulted in some adjustments to the coding framework. Coding was then completed by JD and TE. Rigour was maintained by having an audit trail for the analysis, conducting negative case analysis and regular review of coding to ensure consistency. All authors developed consensus around the final conceptual model which emerged from the data.

## Results

Eighteen participants (labelled T1-T18 in order of interview) had a mean age of 37 years (range 26 to 62 years) and their mean resilience score was high compared to population norms (Table 1). Ten were male, 14 were Australian-born, eight had a rural origin, and 16 were married or partnered with half of the partners relocating to rural areas with the GP registrar.

**Table 1:** Sample characteristics

Demographic feature	Characteristics	Participants
Gender	Male	10
	Female	8
Age	Mean year (range)	37 (26–62)
Australian born	Yes	14
	No	4
Rural origin of GP registrar	Yes	8
	No	10
Marital status	Married/partnered	16
	Single	2
Partner living in rural location	Yes	8
	No	8
With GP registrar	N/A	2
Resilience Scale 26 item^a^	Mean score (range)	154 (129–182)

^a^Population norms (Wagnild 2009). Very low 25–100, low 101–115, moderately low 116–130, moderately high 131–145, high 146–160, very high 161–182

Six main themes emerged from the data describing the GP registrars’ experiences related to resilience and how they sought to manage them or build resilience. The themes included: i) tension between clinical caution versus clinical courage, ii) tension between persistence versus flexibility, iii) tension between reflective practice and task-focussed practice iv) tension between personal connections versus professional commitment; v) skills for balance, and finally vi) support versus stretching by supervisors (Table 2).

**Table 2:** Themes

Theme	Description	Quote
Tension 1: Clinical caution versus clinical courage	To stay safely within one’s current level of professional efficacy or to use first principles and cover ‘red flags’	*I learnt so much doing ICU. They said, ‘oh, it's useless. There’s no ICUs out in the west’. I said, ‘well, I wouldn’t mind doing it anyway’. I tell you what, its just been great. It teaches you how to deal with critically ill people and that’s what you need when you’re going rural. T1*
		*I think some people want everything to come to a neat conclusion immediately, order a test, get a result, know the answer. I think that you have to be happy to not always know what the answer is and know that you may never know.T7*
		*You’ve basically got to step back, try and look at it from a broader view from that and say, right I’ll tackle this with the first principles, you always revert to your first principles, you make sure that you’ve got your red flags covered, your ‘what ifs’. T18*
Tension 2: Flexibility versus persistence	To work persistently towards professional goals while knowing when to re-evaluate and change goals	*So there you need to learn to just let things go. So you learn these skills as time goes by” T16*
		*If something goes wrong and you re-evaluate and go, well that didn’t work, but how are we going to get towards the goal? T5*
Tension 3: Reflective practice versus task-focussed practice	To reflect on own and other experiences while getting the job done	*Practical examples that would make you go into their shoes and feel like, well, you know, this is something that is very very horrible. How would I react if I was put in that situation? I’d probably break down. But what is it about this person that I can learn from and develop their strength, their inner strength to resist that kind of temptation of being worse off than I think I would be?T2*
		*… having that ability to pull your poker face out buys you a lot of time and that you still then react to it appropriately later, but at the time you don’t have the luxury of doing that, … you kind of you do what you have to do and at the end of the day you can sit down and go, wow, that was kind of massive. In scenarios like that everyone seems to talk, which is good because people automatically do that. T4*
Tension 4: Personal connections versus professional commitment	To be sustained by others or to draw meaningfulness from clinical work	*I think really good family support has really helped me. Sometimes if you have had a bad situation at work or sometimes you play it up to more than what it is, talking to family, especially those who are in the field and kind of understand how things tick I’ve found really helpful. T10*
		*Well yeah the rural generalist program is one that really like and it really suits me and in some ways you could say it was designed for me….. and it’s a small little hospital where you had open access for the undifferentiated patients that presents with a problem and it’s got a significant emergency, significant outpatients segment. It’s got obstetrics and a huge indigenous population. It suited me.* *T8*

GP registrars described medical training pressures including: coping with a new role, bureaucratic burdens, and occasional negative feedback. These training issues came on top of clinical pressures including: clinical uncertainty in the GP setting, and dealing with patient distress. GP registrars perceived on the whole that they were resilient however some registrars also acknowledged they were not as resilient as they ideally wanted. When considering the registrar responses, four primary tensions emerged from the data as dichotomous variables. Registrars described attempts to find middle ground, balancing these opposing tensions to maintain a sense of wellbeing.

### Tension 1: Clinical caution versus clinical courage

GP registrars described experiencing a tension between clinical caution (where they deferred to others and constrained their scope of practice) and clinical courage (where they took initiative and pushed boundaries to extend their clinical skills). Clinical caution was often motivated by feeling responsible to ensure quality of care within the registrars’ current level of professional efficacy. One suggestion to build resilience for rural GPs was to develop, use and have confidence in clinical skills considered necessary for rural work, particularly when managing critically ill people. For one GP registrar, Intensive Care training assisted with developing confidence in emergency skills needed for rural areas. The down side of clinical caution was recognised as general practitioners becoming deskilled when acute patients were managed centrally by specialists. In juxtaposition to clinical caution, several rural GP registrars recognised their clinical resilience had developed further with exposure to clinical challenges in rural practice. Registrars described the need to be prepared for surprises in clinical situations and to learn from difficult situations. One interviewee described using cognitive strategies: looking at the clinical situation from a broader view using first principles and covering ‘red flags’. Others described accessing collegial support provided within their practices when they felt it necessary to debrief after experiencing a challenging event and the capacity to telephone local specialists for advice in order to extend the clinical services they provided for their patients.

### Tension 2: Flexibility versus persistence

Registrars reported that medical training requires considerable persistence. They also described dealing with setbacks and challenges by adopting a pragmatic stance while re-evaluating strategies and maintaining some flexibility was useful. Several registrars described situations where they had to change or ‘let go’ of strategies that did not work to reach their professional goals. Letting go was not easy to do. It was only after persisting hard to achieve a goal that registrars came to reassess the situation as futile and there was little point continuing their action. For example, one registrar [T14] described wanting to make changes in the health system for rural people with suicidal ideation but realised that despite her efforts she could not improve access to care and chose to accept the status quo in her short term placement. Another way of dealing with challenges was to set a time marker (for example, end of year) and then re-assess the situation. In that way, registrars described persistence, by giving the situation time, but also flexibility in knowing they would make a decision in the future. Another strategy was to simply have a break before dealing with the challenging circumstances again.

### Tension 3: Reflective practice versus task-focussed practice

Registrars described two skills which they traded off one against the other: taking the time to reflect on experiences, and a pragmatic use of time focussed on completing the task at hand. Registrars recognised advantages of reflecting on their own experiences, and those of their patients and professional peers. For example walking in patients’ shoes and reflecting on patient’s lives, GP registrars described the potential for personal learning through practical examples of their patients’ resilience. Reflecting also on situations where other GPs or registrars had “fallen apart”, they described being able to determine what they needed to put in place to protect themselves in other challenging situations. In contrast to reflective practice, some registrars described strength from the ability to detach from the emotion of clinical events and get the job done. One registrar would “pull your poker face” [T4] during challenging events, deferring reflective practice until later. Using black humour was reported to relieve tension in very stressful situations. This detachment however was also seen as a potential weakness. It was felt important to identify one’s emotions after a challenging situation (“am I angry, am I sad, am I frustrated”) [T4], suggesting that emotion should be “dealt with” on the day it happens.

### Tension 4: Personal connections versus professional commitment

GP registrars in this study prioritized work-life balance. GP registrars identified that surrounding themselves with family and friends for personal support and encouragement helped them maintain resilience. This was particularly important when coping with challenging situations at work or when they felt they were struggling personally. Resilience was challenged when rural registrars lived away from immediate family. When managing an ongoing challenging situation, these registrars could maintain resilience by scheduling ahead activities that made them feel good, for example visiting family in the city. On the other hand GP registrars described the personal rewards of professional commitment including setting goals and prioritizing time and energy towards reaching those goals. This focus gave GP registrars an opportunity to acknowledge their achievements, before setting other goals. Resilience was strengthened by success and by taking the opportunity to re-assess professional goals when they were challenged, including setting limits around workload and work demands (for example on call hours).

Rural GP registrars identified several personal skills which supported their clinical resilience, in addition to recognising the role of their supervisors (Table 3).

**Table 3:** Resolving the tensions

Theme	Description	Quote
Personal skills for balance	Meaningfulness	*Yeah. So I suppose GP is the cradle to grave care. That’s what I like. I like the continuity of care and I like knowing the community, which is a general practice kind of thing. T7*
	Self reflection for capacity to be resilient	*..allow resilience to be lived and then reflection on those experiences can allow resilience to be recognised. T8*
	Optimistic attitude	*The confidence to do it was important. I think one of those kinds of attributes would be just maybe self confidence and trust and belief that it will work. T2*
Supervisor support and stretching	Clinician supervisors take an active role in supporting and pushing registrars	*To teach resilience you put people in those positions where they have a lot of responsibility but you give them the support. … if you were there and you don’t know what to do then you can get a hold of somebody who does know what to do and who will come in. T3*

### Skills for balance

Registrars described improved resilience with good self-care including: sufficient sleep, exercise and leisure activity to balance out the demands of professional life. Registrars identified several internal processes which supported resilience by enabling them to balance the tensions described above: an optimistic attitude; self-reflection and meta-cognition. Registrars who reported being strongly positive about their work frequently described the meaningful nature of caring for a community. It was reported as important to recognise when resilience was getting low and then to identify stressors that challenged resilience. Self-reflection was also recognised as a powerful tool to identify one’s capacity for resilience and to learn and grow from experiencing adversity. Registrars suggested that reflecting upon challenging experiences helped identify their personal capacity and allowed them to be realistic about workloads and responsibilities. Registrars described seeking to step back and view the situation from afar and put mental structures in place to manage stressors. Examples of these metacognition techniques given included: breaking life up into managing a day at a time so they do not get overwhelmed by the big picture, not catastrophizing about problems; and reframing difficult situations as temporary.

### Supervisor support and stretching

Registrars new to rural areas reported facing challenges without their usual professional and personal supports. While the tensions outlined above describe what issues GP registrars were managing in order to have resilience, this theme describes how GP supervisors engaged in supporting the registrars. Having support was recognised as important to build resilience in rural practice and registrars described the value of: backup clinical support at work; professional mentorship (included debriefing/talking to others) and social support in rural settings (help in developing registrars’ local personal networks). One registrar [T5] suggested that “rural practice life coaches” could help build resilience, by advice on goal setting and managing setbacks. In contrast it was acknowledged that exposure to challenging experiences and ‘not having things easy’ (in medical practice, and in life in general), plays an important role in personal learning of how to cope with those experiences. Being thrown ‘in the deep end’ was discussed in relation to scenario-based training. Confidence and resilience were developed by practicing difficult or complex clinical skills.

## Discussion

Resilience is a continuous dynamic process of dampening the impact of challenges and learning from them to maintain or improve wellbeing. In this study, GP registrars reported they were resilient, basing their judgement on having dealt with challenging situations in the past. This belief is reflected in this sample’s high mean score on the Resilience Scale which corresponded to mean scores in the larger study from which this sample is drawn [9, 12]. The study sample included a high number of older GP registrars as doctors on the ACRRM Independent Pathway tend to be more experienced clinicians. Age and being partnered are factors known to have a positive correlation with resilience scores [13]. Although we have not been able to find evidence in the literature linking resilience with retention of rural GPs, it is not unreasonable to assume a relationship between these factors as there is clear evidence of the reverse – that burnout correlates with high intention to leave rural practice [14].

**Fig. 1:**
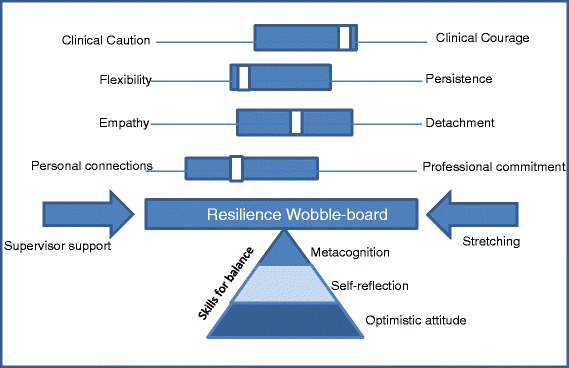
Resilience wobble-board model. Managing tensions through optimistic attitude, self-reflection and metacognition with medical education providing support and stretching

This paper found a number of themes which provide a conceptual model of resilience in rural Australian GP registrars (Fig. 1). The registrars in this study described four discrete tensions which they juggle in order to maintain balance in their professional life. Resilience is maintained while these tensions remain within acceptable limits. This study found that the balance between tensions is unique to each individual, and that the acceptable limits for each tension can be expanded through personal learning. The authors conceptualised this as each registrar having their own individual resilience ‘wobble-board’ which they need to maintain in balance using their personal skills with the assistance of support and stretching from others. The stability of the board can be increased with experience as registrars can tolerate greater variations in tensions. It is likely that this model is generalizable internationally in rural GP training contexts. The tensions experienced by rural GP registrars may not be unique to the rural context, and so this model may be further generalizable to urban general practice training programs.

The tension between clinical caution and clinical courage has been eluded to previously in the literature with traditional tertiary hospital medical training enculturating junior doctors to respect senior specialists in a hierarchical system to the potential detriment of their clinical initiative [13]. This ‘learned helplessness’ has been recognised as a disincentive to recruitment to rural practice [15]. Intolerance of clinical uncertainty has previously been demonstrated to be associated with burnout in Australian GP registrars while concern for bad outcomes correlated more strongly for doctors dealing primarily with emergency presentations [2, 3]. Clinical courage, recognises that evidence-based medicine is frequently context void and that rural and remote practice seeks to balance this with clinical uncertainties of patient-centred care where the likelihood of adverse outcomes are undetermined [16]. Medical educators have proposed that resilience could be facilitated by supporting registrars to embrace challenging clinical situations as opportunities for growth [2, 17]. Certainly higher novelty seeking and lower harm avoidance personality attributes are more common in GPs engaged in rural practice [18]. This study demonstrates that balancing clinical caution with clinical courage is a key dynamic in determining resilience of rural registrars.

Balancing persistence with flexibility was found to affect rural GP registrar resilience in this study. Certainly both rural and urban Australian general practitioners have significantly higher mean levels of persistence in comparison to population norms [18, 19]. Importantly persistence in rural doctors has been shown to correlate with resilience [9]. In contrast to this finding, resilience in experienced German physicians has been linked to the capacity to recognise when change was necessary [20]. Perhaps recognising the negative outcomes of unfettered persistence, Longenecker has previously proposed resilience of rural doctors-in-training may be improved through incorporating the pursuit of adaptability into rural training curricula [17].

Reflective practice and clinician empathy are attributes worth cultivating in GP registrars as they can protect against burnout and positively correlate with improved patient outcomes [2, 21]. However empathy is complex in rural areas where overlapping relationships in rural communities often result in blurred therapeutic boundaries between caregivers, patients and families [22]. Detachment, particularly task-focussed practice, may allow registrars to manage themselves in traumatic clinical situations [23]. Task-focussed practice may also assist resilience through conscious management of proximity and professional boundaries in the patient—doctor relationships [20, 24]. GP training organisations have a responsibility to mentor registrars to develop adaptive strategies to become reflective practitioners and empathise with their patients while managing complex relationships in different spheres of community.

GP registrars in this study prioritized balance between their personal connections and professional commitment. Achieving work-life balance has previously been demonstrated to be a significant source of stress for GP registrars [25]. Resilience has long been recognised to be improved through meaningful personal connections and resilience in doctors is no exception [1, 20]. Secure attachments allow individuals to set future goals operating from a secure base [1, 26]. Work commitments and focus on career goals can constrain personal connections for early career doctors, however resilience is also improved by the personal meaning through patient care and the value registrars place on their clinical role [2, 10, 11, 20]. We recommend that GP training organisations have a role in mentoring registrars to engender a strong sense of professional identity and intellectual engagement in rural practice, while developing boundaries around work in order to invest in mutually supporting personal relationships outside medicine [11, 27].

Our study findings concur with previous studies which indicate GP registrars with high resilience accessed professional and personal supports to deal with challenges [20, 28]. This study also demonstrated that individuals can be stretched by supervisors to expand their limits of comfort at managing the key tensions. This finding is consistent with Mezirow’s Transformative Learning Theory which recognises that taking people to their ‘edge of knowing’ can result in growth, where adult learners also have the autonomy to adapt and an effective peer group to provide support [29].

Registrars reported that their ability to manage clinical tensions in rural GP training was dependent on them having or acquiring skills for balance including: an optimistic attitude, self-reflection skills and metacognition. Resilience has been previously linked to a positive attitude towards life, hope and a psychological sense of mastery [1, 30]. Optimism can be learned through modelling and cognitive behavioural techniques, and sense of mastery develops as a learners gain experience in rural practice [31]. Resilience is enhanced in doctors who have personal reflexivity skills [20, 32]. Longernecker’s paper indicates that GP training should prioritise the development of self-reflection as “a skill and a habit” [17].

### Limitations

The study has some methodological limitations. All study participants were currently working in rural practice and so it is not clear if the study findings are unique to rural general practice. Participants self-selected which may bias the sample towards those who are resilient. However, this bias more likely benefits the findings when developing a theoretical framework to describe the development of resilience in GP registrars. GP registrars may have given socially desirable responses and it may be unrealistic to expect GP registrars to fully consider resilience within the time given in the interview.

## Conclusion

The clinical resilience wobble-board provides a model for developing and maintaining a stable resilient base to manage professional and personal challenges in rural GP registrars. Building and maintaining this resilience base requires a balance between: clinical caution and courage, personal flexibility and persistence; reflective practice and task-focused practice and work life balance. Educators should formally assist GP registrars to recognise and reflect on these tensions with an optimistic attitude and enhance their metacognition skills to face these challenges and extend resilience as a core competency for rural GP training.
